# Addendum: Big data driven perovskite solar cell stability analysis

**DOI:** 10.1038/s41467-024-48894-x

**Published:** 2024-06-05

**Authors:** Zhuang Zhang, Huanhuan Wang, T. Jesper Jacobsson, Jingshan Luo

**Affiliations:** 1 Institute of Photoelectronic Thin Film Devices and Technology, Solar Energy Research Center, Key Laboratory of Photoelectronic Thin Film Devices and Technology of Tianjin, Ministry of Education Engineering Research Center of Thin Film Photoelectronic Technology, Renewable Energy Conversion and Storage Center, Nankai University, 300350 Tianjin, China; 2 Haihe Laboratory of Sustainable Chemical Transformations, 300192 Tianjin, China

**Keywords:** Solar cells, Solar cells

Addendum to: *Nature Communications* 10.1038/s41467-022-35400-4, published online 10 December 2022

In the original version of the article, we developed a set of heuristics for comparing stability data, which was used to analyze the dataset from the Perovskite Database Project. After the initial publication, we received feedback from a reader and identified some errors in the original dataset. To ensure that those errors had no major influence on the conclusion, we checked the data carefully and rerun the analysis with the corrected data. The detailed results are listed below.

1. The errors in the data we used to draw our conclusions come from the errors in the original database. Those existed when we downloaded the data from the Perovskite Database, and none of our operations (stability data extraction, statistical analysis, “data.m” file generation) changed the original data. After a careful check, we found 95 errors in the 7419 data points we used. Those are listed in the attached file, “data correction.xlsx”, and are primarily concerned with the “Cell_architecture” parameter, i.e. mostly either nip or pin.
2. After correcting the errors, we rerun the analysis. The new results show negligible changes. This is what to be expected as only a small fraction of the dataset had the wrong cell_architecture label.

For the results related to the device architecture, the data correction resulted in small shifts in the average and variances and a slightly larger shift in the T_A_/T_B_ ratios. The analysis of the categories with more data, i.e. nip and pin (Tables 1 and 2) were less affected by the data correction, than those with fewer samples, e.g., inorganic HTL and doped organic HTL (Tables 3 and 4), which illustrates the importance of large dataset. There were no changes in the results for analyses not based on a separation of devices based on cell architecture, such as the analysis of the importance of the tolerance factor (Tables 5 and 6). To summarize, the data correction did not result in any changes in the conclusions.

**Table 1:** Statistical results of n-i-p, spiro-based n-i-p, and p-i-n structured devices. (Before data correction)

Group	Sample	Average	Variance	Size	*H*_0_ accepted	*μ*	*T*_A_/*T*_B_ ratio
1	N-i-p structured w/o encapsulation	4.879	4.921	3808	Yes	0.211	1.235
	P-i-n structured w/o encapsulation	4.557	5.390	1596			
2	Spiro-based N-i-p structured w/o encapsulation	4.692	4.576	2887	Yes	0.021	1.022
	P-i-n structured w/o encapsulation	4.557	5.390	1596

**Table 2:** Statistical results of n-i-p, spiro-based n-i-p, and p-i-n structured devices. (After data correction)

Group	Sample	Average	Variance	Size	*H*_0_ accepted	*μ*	*T*_A_/*T*_B_ ratio
1	N-i-p structured w/o encapsulation	4.875	4.933	3819	Yes	0.205	1.227
	P-i-n structured w/o encapsulation	4.560	5.386	1577			
2	Spiro-based N-i-p structured w/o encapsulation	4.689	4.578	2910	Yes	0.016	1.016
	P-i-n structured w/o encapsulation	4.560	5.386	1577

**Table 3:** Statistical results of devices with different HTLs and electrodes. (Before data correction)

Group	Sample	Average	Variance	Size	*H*_0_ accepted	*μ*	*T*_A_/*T*_B_ ratio
1	Undoped organic HTL	5.028	4.668	341	Yes	0.043	1.044
	Doped organic HTL	4.775	5.036	3074			
2	Inorganic HTL	5.854	7.219	57	Yes	0.583	1.791
	Doped organic HTL	4.775	5.036	3074			
3	HTL/Carbon	7.132	5.745	94	Yes	1.969	7.163
	Doped organic HTL	4.775	5.036	3074			
4	HTL-free/Carbon	6.458	4.261	257	Yes	1.444	4.240
	Doped organic HTL	4.775	5.036	3074

**Table 4:** Statistical results of devices with different HTLs and electrodes. (After data correction)

Group	Sample	Average	Variance	Size	*H*_0_ accepted	*μ*	*T*_A_/*T*_B_ ratio
1	Undoped organic HTL	5.034	4.698	337	Yes	0.048	1.049
	Doped organic HTL	4.774	5.038	3104			
2	Inorganic HTL	5.995	8.105	49	Yes	0.687	1.988
	Doped organic HTL	4.774	5.038	3104			
3	HTL/Carbon	7.192	5.813	96	Yes	2.034	7.645
	Doped organic HTL	4.774	5.038	3104			
4	HTL-free/Carbon	6.467	4.203	258	Yes	1.455	4.283
	Doped organic HTL	4.774	5.038	3104

**Table 5:** Statistical results of devices with different tolerance factor regions. (Before data correction)

Group	Sample	Average	Variance	Size	*H*_0_ accepted	*μ*	*T*_A_/*T*_B_ ratio
1	Large (>0.95)	5.300	5.362	1691	Yes	0.619	1.858
	Medium (0.85–0.95)	4.574	4.609	3608			
2	Small (<0.85)	6.049	8.097	402	Yes	1.282	3.605
	Medium (0.85–0.95)	4.574	4.609	3608

**Table 6:** Statistical results of devices with different tolerance factor regions. (After data correction)

Group	Sample	Average	Variance	Size	*H*_0_ accepted	*μ*	*T*_A_/*T*_B_ ratio
1	Large (>0.95)	5.300	5.362	1691	Yes	0.619	1.858
	Medium (0.85–0.95)	4.574	4.609	3608			
2	Small (<0.85)	6.049	8.097	402	Yes	1.282	3.605
	Medium (0.85–0.95)	4.574	4.609	3608			


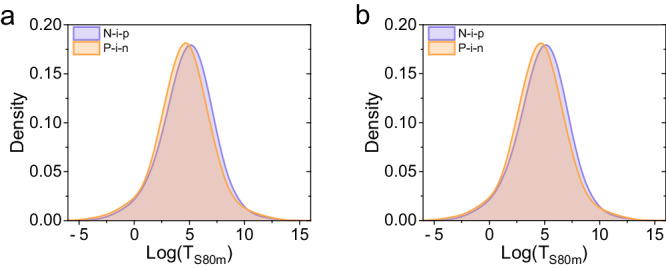


**Fig. 1**. The kernel density estimation of the log(T_S80m_) values for n-i-p and p-i-n structured devices. **a** Before data correction. **b** After data correction.


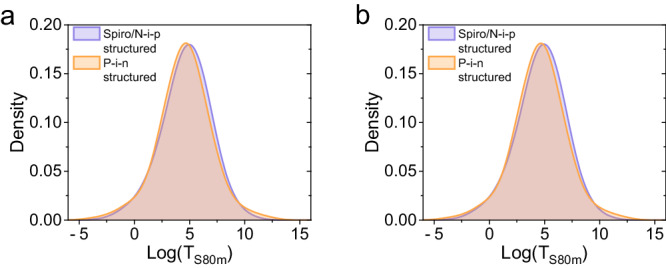


**Fig. 2**. The kernel density estimation of the log(T_S80m_) values for spiro-based n-i-p structured devices and p-i-n structured devices. **a** Before data correction. **b** After data correction.


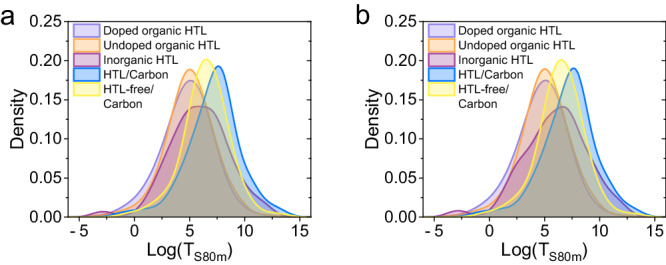


**Fig. 3**. The kernel density estimation of the log(T_S80m_) values for devices with different HTLs and electrodes. **a** Before data correction. **b** After data correction.


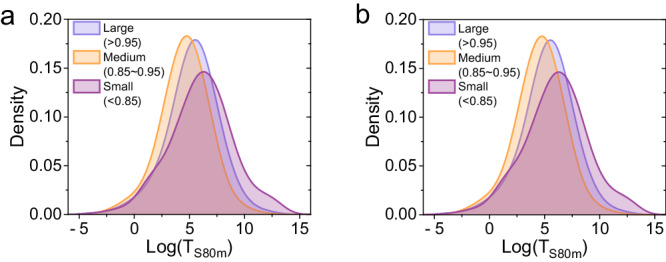


**Fig. 4**. The kernel density estimation of the log(T_S80m_) values for devices with different tolerance regions. **a** Before data correction. **b** After data correction.

3. A small number of errors in a large dataset is to be expected, especially for datasets based on manual human data extraction which is the case in the Perovskite Database. Moreover, a small amount of errors in a dataset usually does not change the validity of a statistical analysis more than somewhat widening the error bars. That is one of the advantages of big data. Errors are not limited to databases of device metrics, as also all experimental data have noise. Noise in a large heterogeneous dataset does usually not have a significant influence on the results as the errors average out each other and are dwarfed by the rest of the data.

To sum up, our work focuses on the development of a set of heuristics that can be used for a rough comparison of stability data. A small number of errors in the dataset used for the analysis does not negate the meaning and the value of this work. When a few errors were found in the original dataset, the question was raised whether or not this had affected the conclusions drawn from our analysis. We then checked and corrected the data and found that this did not influence the reliability of our analysis or the conclusions that were drawn from it. If something, this extra check supports the robustness of the analysis.

