# Supplementary material for: Big data driven perovskite solar cell stability analysis
Source: Nat Commun. 2022 Dec 10;13:7639. doi: 10.1038/s41467-022-35400-4 (PMC9741627; doi:10.1038/s41467-022-35400-4)
Supplement: Supplementary file 1 — Supplementary Information [file 41467_2022_35400_MOESM1_ESM.pdf]

Supplementary Information

Big Data Driven Perovskite Solar Cell Stability Analysis

Zhang et al.

Supplementary Notes

Supplementary Note 1: The calculation of T_{S80}

For T_{S80}, the value is directly used if provided, otherwise we use a double exponential decay function to calculate the T_{S80} with other parameters provided in the database including E_{1000h}, T_{80} and the time and efficiency at the end of the experiment (T_{end}, E_{end}). It is proved that the double exponential decay function can accurately fit the efficiency decay curve[1] and the function is given by:

$$\eta = A_1 \cdot e^{-\frac{t}{\tau_1}} + A_2 \cdot e^{-\frac{t}{\tau_2}} \tag{1}$$

where η represents the efficiency at time t, A_1 and A_2 are the coefficients of the burn-in and degradation regions, τ_1 and τ_2 are the time constants of the two regions. Because the burn-in region is generally a short period, we assume (1000h, E_{1000h}), (T_{80}, 80%), and (T_{end}, E_{end}) points are all in the slow degradation region and the burn-in part is neglected in the function. We obtain:

$$\eta = A_2 \cdot e^{-\frac{t}{\tau_2}} \tag{2}$$

and the points (1000h, E_{1000h}), (T_{80}, 80%) and (T_{end}, E_{end}) are substituted into the function to calculate A_2 and τ_2. Assuming the fitted value at time zero approximates the stabilized efficiency at the end of the burn-in region as Figure 1b shows, T_{S80} is obtained by:

$$\eta(T_{S80}) = A_2 \cdot e^{-\frac{T_{S80}}{\tau_2}} = A_2 * 80\% \tag{3}$$

and

$$T_{S80} = -\ln 0.8 \cdot \tau_2 \tag{4}$$

Supplementary Note 2: The detailed description of hypothesis test method

Assuming two normal-distributed samples named sample A and sample B with different strategies, a variable t_0 is given by:

$$t_0 = \frac{\overline{X_\mathrm{A}} - \overline{X_\mathrm{B}} - (\mu_\mathrm{A} - \mu_\mathrm{B})}{S_\mathrm{w}\sqrt{\frac{1}{n_\mathrm{A}} + \frac{1}{n_\mathrm{B}}}} \tag{10}$$

and S_w is given by:

$$S_\mathrm{w} = \sqrt{\frac{(n_\mathrm{A} - 1)S_\mathrm{A}^2 + (n_\mathrm{B} - 1)S_\mathrm{B}^2}{n_\mathrm{A} + n_\mathrm{B} - 2}} \tag{11}$$

where $\overline{X_\mathrm{A}}$ and $\overline{X_\mathrm{B}}$ are averages of the two samples, μ_A and μ_B are averages of the two populations which the two samples come from, n_A and n_B are sizes of the two samples, S_A^2 and S_B^2 are variances of the two samples. The variable t_0 follows a student's t distribution, whose probability density distribution function is given by:

$$\mathrm{p}(x, n) = \frac{\Gamma\left[\frac{n+1}{2}\right]}{\sqrt{\pi n}\,\Gamma\left[\frac{n}{2}\right]}\left(1 + \frac{x^2}{n}\right)^{-\frac{(n+1)}{2}}, -\infty < x < +\infty \tag{12}$$

where x is the variable value, n is the parameter of the distribution, $\mathrm{p}(x, n)$ is the probability density and the distribution is donated by $\mathrm{t}(n)$. The cumulative distribution function of $\mathrm{t}(n)$ is given by:

$$\mathrm{F}(x, n) = \int_{-\infty}^{x} \mathrm{p}(x, n)\, dx \tag{13}$$

For the distribution of the variable t_0, the parameter n equals $n_\mathrm{A}+n_\mathrm{B}-2$.

Then, we propose two hypothesizes, which are:

$$H_0: \mu_\mathrm{A} \geq \mu_\mathrm{B}, \quad H_1: \mu_\mathrm{A} < \mu_\mathrm{B}$$

where H_0 means the average of the population of sample A is not less than sample B and H_1 is the opposite. For student's t distributed variable t_0, assume $\mu_\mathrm{A} = \mu_\mathrm{B}$ at first and get a variable t_1:

$$t_1 = \frac{\overline{X_\mathrm{A}} - \overline{X_\mathrm{B}}}{S_\mathrm{w}\sqrt{\frac{1}{n_\mathrm{A}} + \frac{1}{n_\mathrm{B}}}} \tag{14}$$

For t_1, if there is:

$$\mathrm{F}(t_1, n_\mathrm{A} + n_\mathrm{B} - 2) = \int_{-\infty}^{\mathrm{t}_1} \mathrm{p}(x, n_\mathrm{A} + n_\mathrm{B} - 2)\, dx \geq 0.95 \tag{15}$$

we can accept H_0 with 95% confidence.

Finally, to what extent the average of the population of sample A is larger than sample B can also be determined by the student's t-test. Assume $\mu_\mathrm{A}-\mu_\mathrm{B}=\mu$ and get a variable t_2:

$$t_2 = \frac{\overline{X_\mathrm{A}} - \overline{X_\mathrm{B}} - \mu}{S_\mathrm{w}\sqrt{\frac{1}{n_\mathrm{A}} + \frac{1}{n_\mathrm{B}}}} \tag{16}$$

When:

$$\mathrm{F}(\mathrm{t}_2, n_\mathrm{A} + n_\mathrm{B} - 2) = \int_{-\infty}^{x} \mathrm{p}(t_2, n_\mathrm{A} + n_\mathrm{B} - 2)\, dx = 0.95 \tag{17}$$

we can just accept the hypothesis μ_A-$\mu_B \geq \mu$ with 95% confidence and the value of μ is the maximum acceptable value of the average difference between two populations. For T_{S80m} values of population A and population B, T_A and T_B, there is:

$$\frac{T_A}{T_B} = e^{\log(T_A)-\log(T_B)} \geq e^{\mu} \tag{18}$$

which means the average decay time of population A is e^{μ} as large as B.

Supplementary Figures

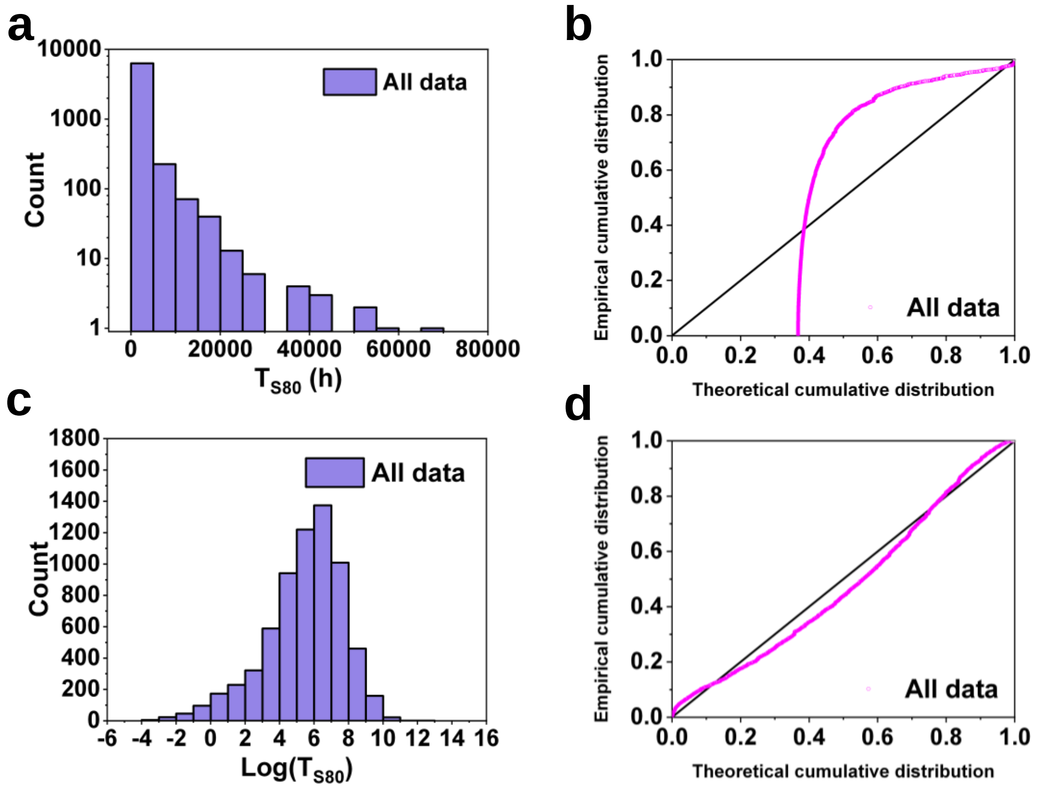

Supplementary Fig. 1 The distribution of T_{S80}. **a-b** The histogram and the normal probability plot of T_{S80} values. **c-d** The histogram and the normal probability plot of log(T_{S80}) values.

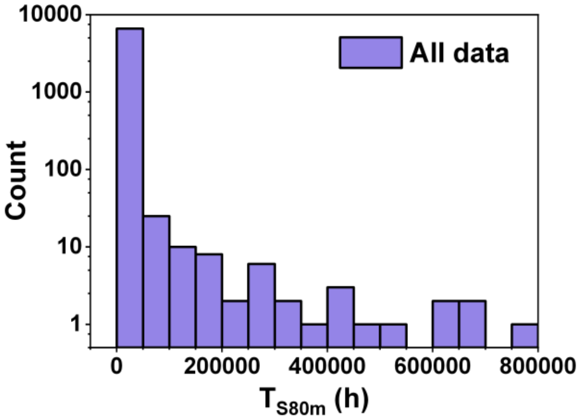

Supplementary Fig. 2 The histogram of T_{S80m} values for all data.

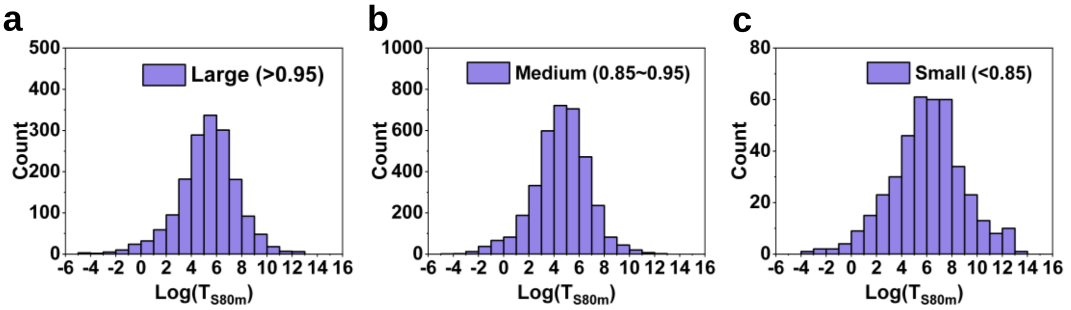

Supplementary Fig. 3 The histograms of log(T_{S80m}) values for different tolerance factor regions of 3D perovskite devices without encapsulation. **a** Large tolerance factors (>0.95). **b** Medium tolerance factors (0.85~0.95). **c** Small tolerance factors (<0.85).

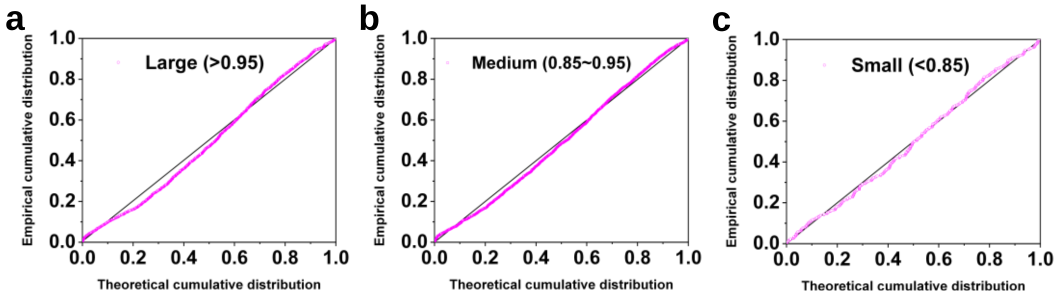

Supplementary Fig. 4 The normal probability plots of log(T_{S80m}) values for different tolerance factor regions of 3D perovskite devices without encapsulation. **a** Large tolerance factors (>0.95). **b** Medium tolerance factors (0.85~0.95). **c** Small tolerance factors (<0.85).

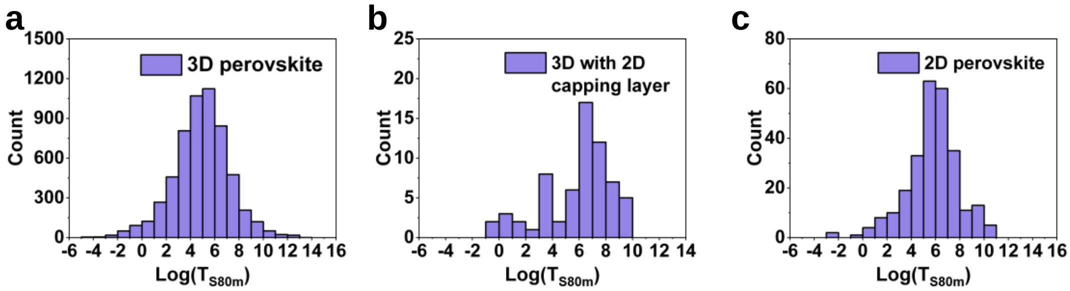

Supplementary Fig. 5 The histograms of log(T_{S80m}) values for devices with different perovskite absorber layers without encapsulation. **a** 3D perovskite. **b** 3D perovskite with 2D capping layer. **c** 2D perovskite.

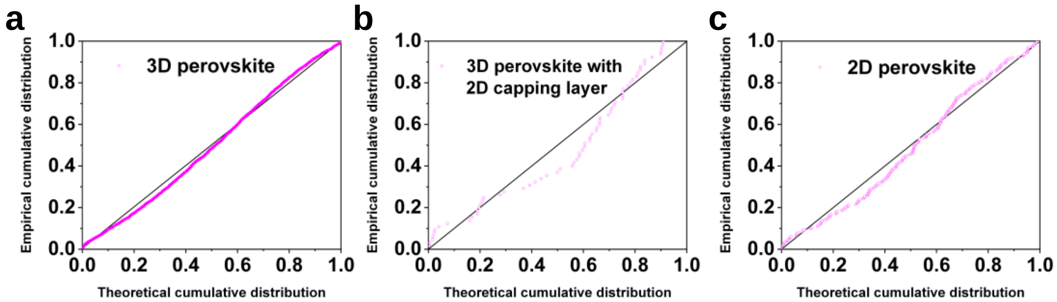

Supplementary Fig. 6 The normal probability plots of log(T_{S80m}) values for devices with different perovskite absorber layers without encapsulation. **a** 3D perovskite. **b** 3D perovskite with 2D capping layer. **c,** 2D perovskite.

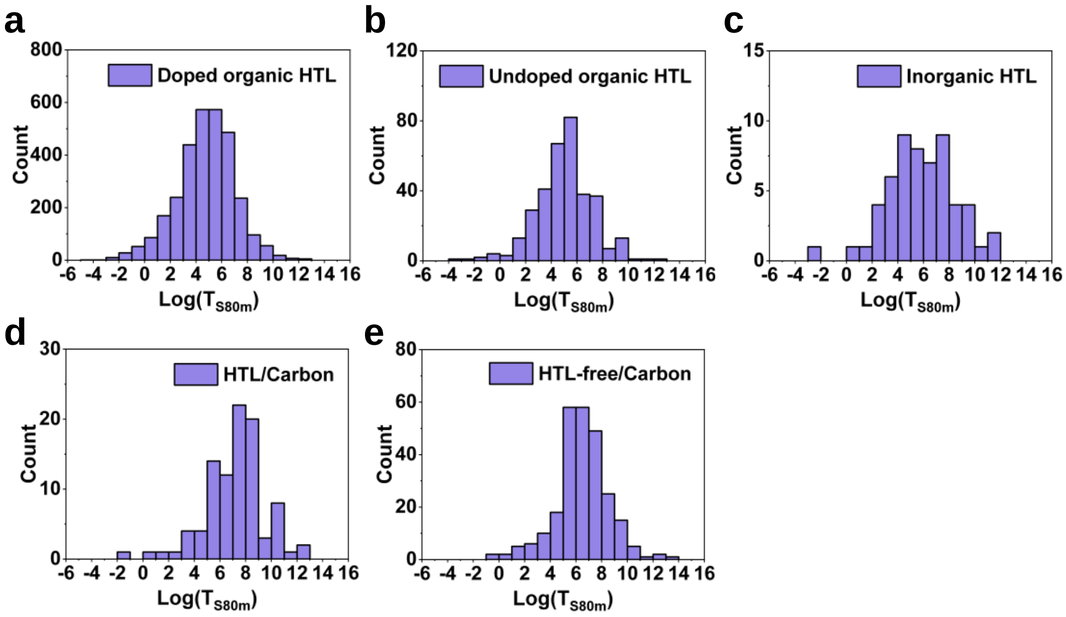

Supplementary Fig. 7 The histograms of log(T_{S80m}) values for devices with different HTLs without encapsulation. **a** doped organic HTL. **b** undoped organic HTL. **c** inorganic HTL. **d** HTL/Carbon. **e** HTL-free/Carbon.

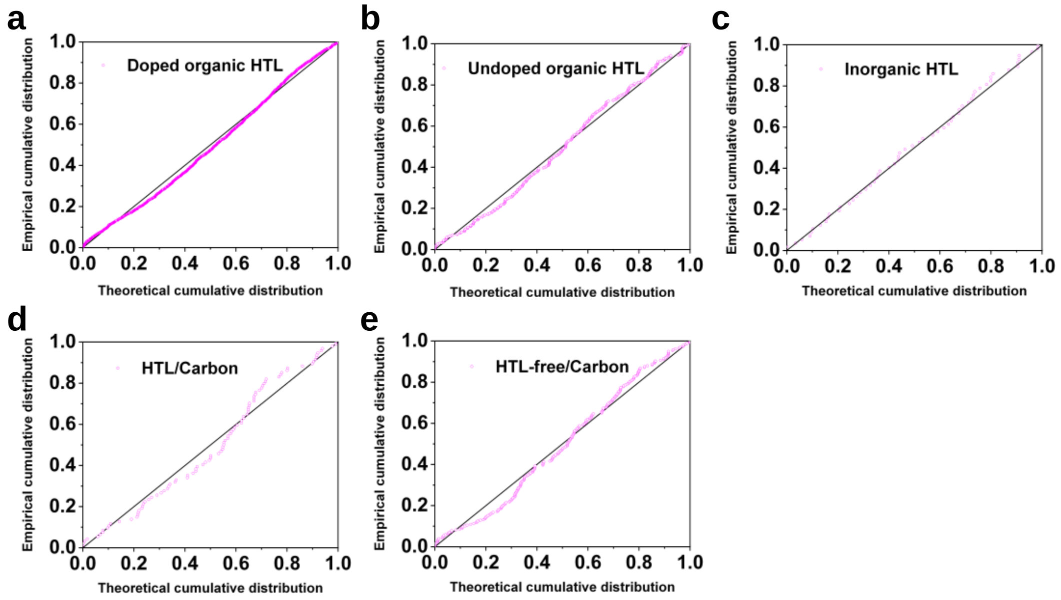

Supplementary Fig. 8 The normal probability plots of log(T_{S80m}) values for devices with different HTLs without encapsulation. **a** doped organic HTL. **b** undoped organic HTL. **c** inorganic HTL. **d** HTL/Carbon. **e,** HTL-free/Carbon.

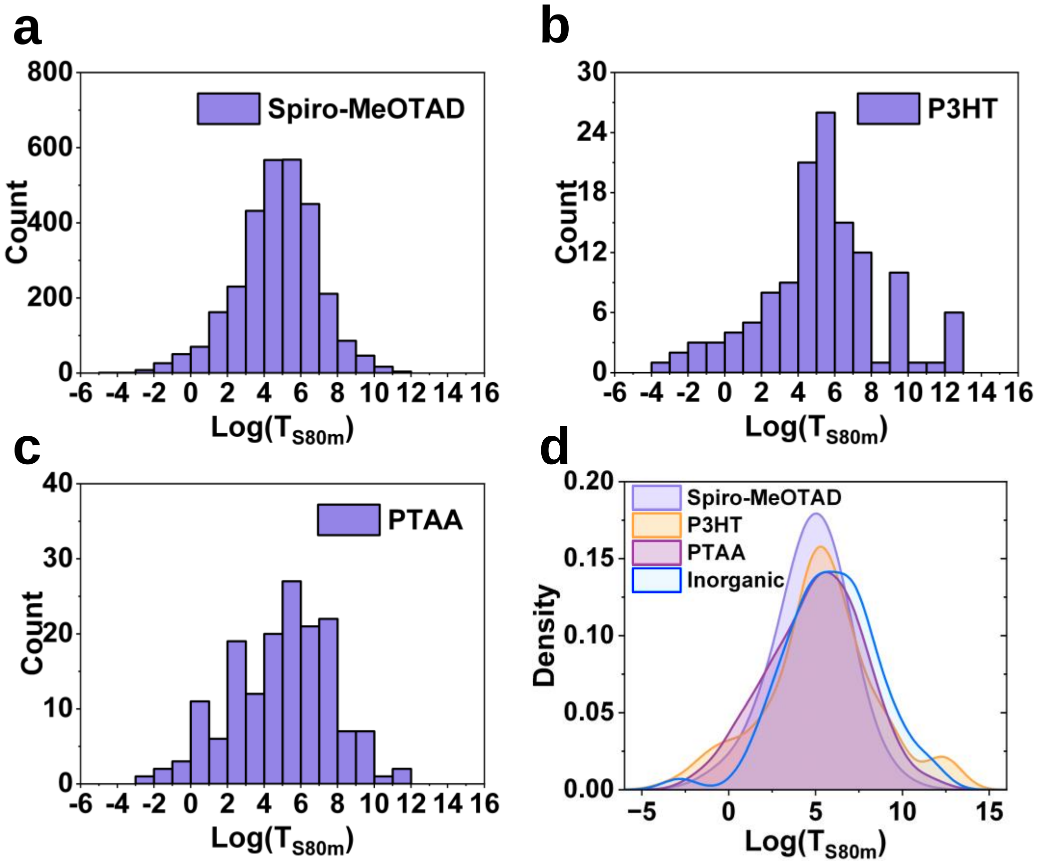

Supplementary Fig. 9 The histograms and kernel density estimation of log(T_{S80m}) values for devices with different HTLs without encapsulation. **a** Spiro-MeOTAD. **b** P3HT. **c** PTAA. **d** kernel density estimation.

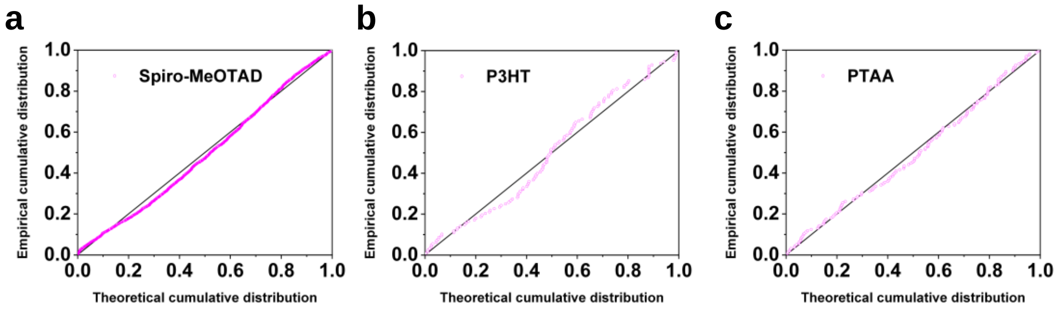

Supplementary Fig. 10 The normal probability plots of log(T_{S80m}) values for devices with different HTLs without encapsulation. **a** Spiro-MeOTAD. **b** P3HT. **c** PTAA.

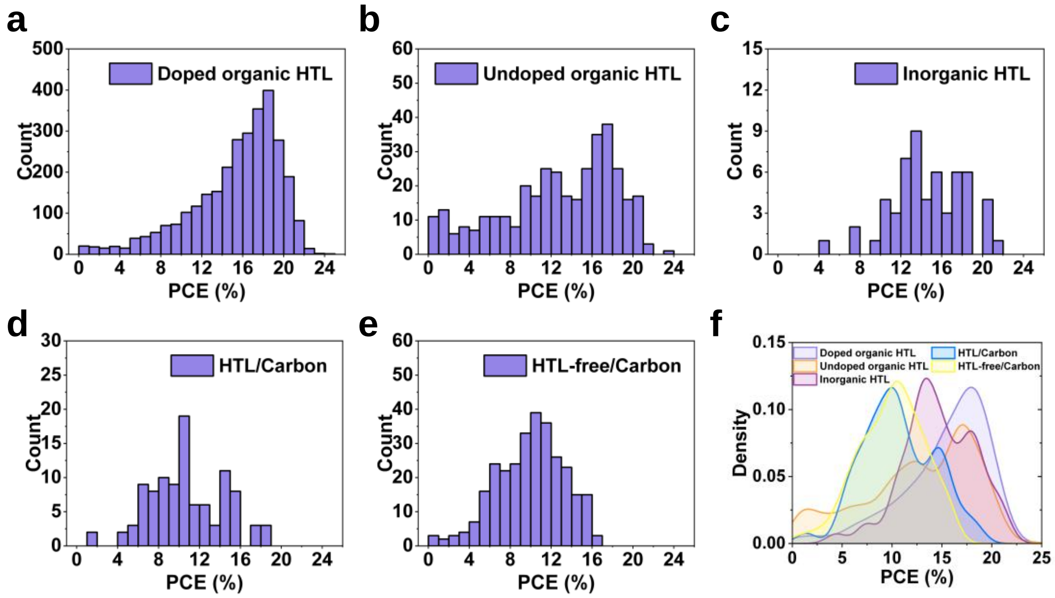

Supplementary Fig. 11 The histograms and kernel density estimation of efficiency values for devices with different HTLs without encapsulation. **a** doped organic HTL. **b** undoped organic HTL. **c** inorganic HTL. **d** HTL/Carbon. **e** HTL-free/Carbon. **f** kernel density estimation.

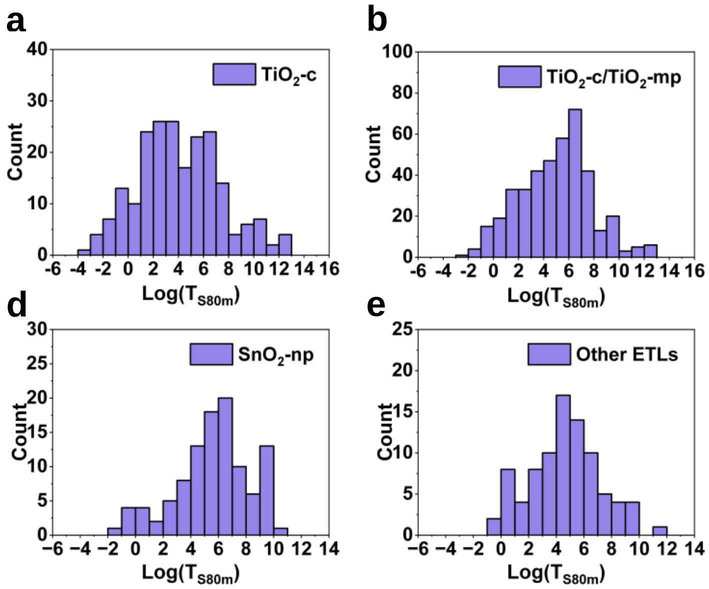

Supplementary Fig. 12 The histograms of log(T_{S80m}) values for devices with different ETLs without encapsulation. **a** TiO_2-c. **b** TiO_2-c/TiO_2-mp. **c** SnO_2-c. **d** SnO_2-np. **e** Other ETLs.

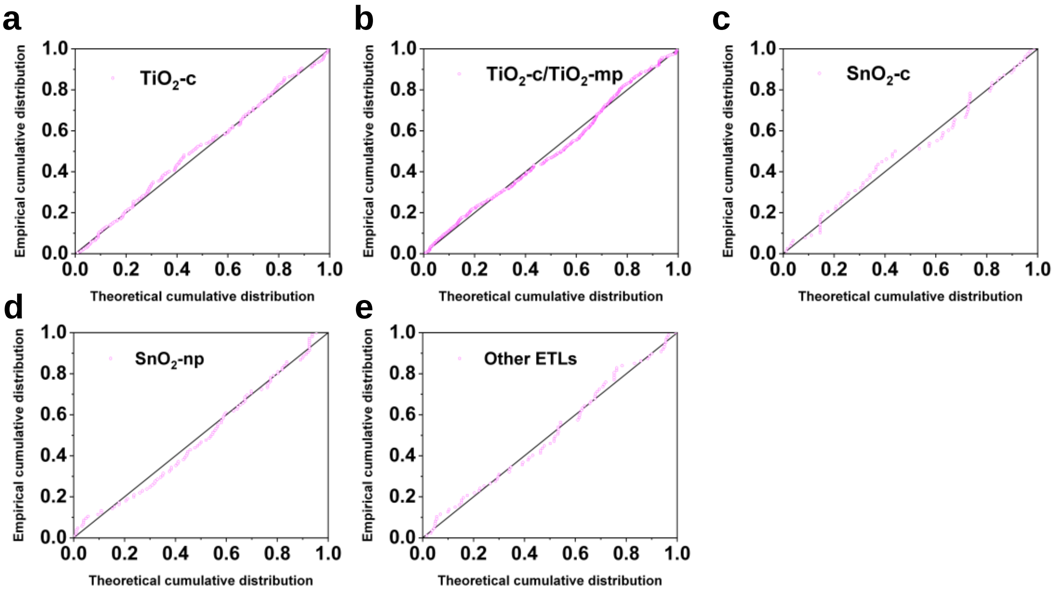

Supplementary Fig. 13 The normal probability plots of log(T_{S80m}) values for devices with different ETLs without encapsulation. **a** TiO_2-c. **b** TiO_2-c/TiO_2-mp. **c** SnO_2-c. **d** SnO_2-np. **e** Other ETLs.

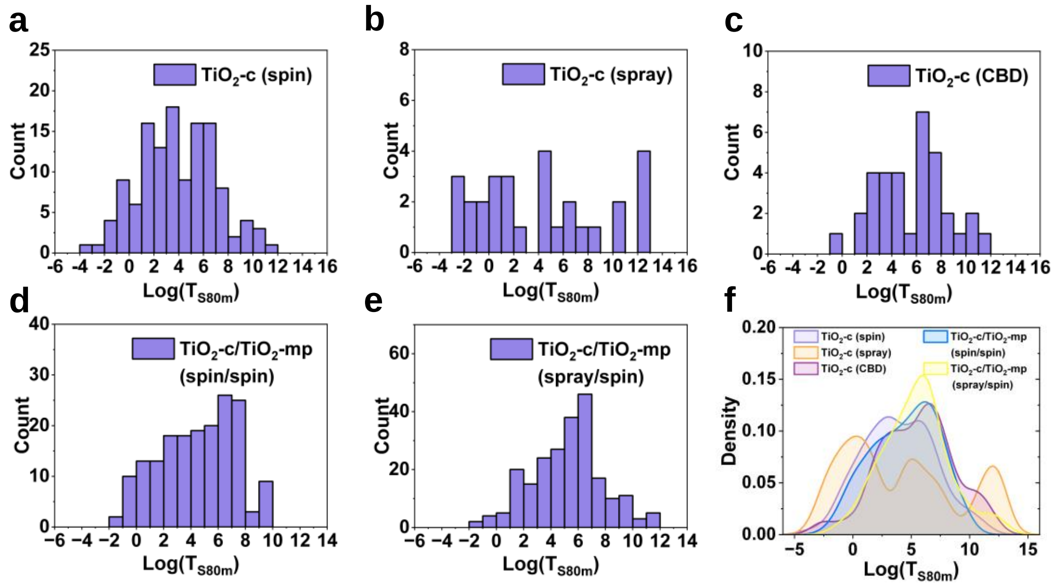

Supplementary Fig. 14 The histograms and kernel density estimation of efficiency values for devices with different HTLs without encapsulation. **a** TiO_2-c by spin-coating. **b** TiO_2-c by spray-pyrolysis. **c** TiO_2-c by CBD. **d** TiO_2-c/ TiO_2-mp by spin-coating/ spin-coating. **e** TiO_2-c/ TiO_2-mp by spray-pyrolysis/ spin-coating. **f** kernel density estimation.

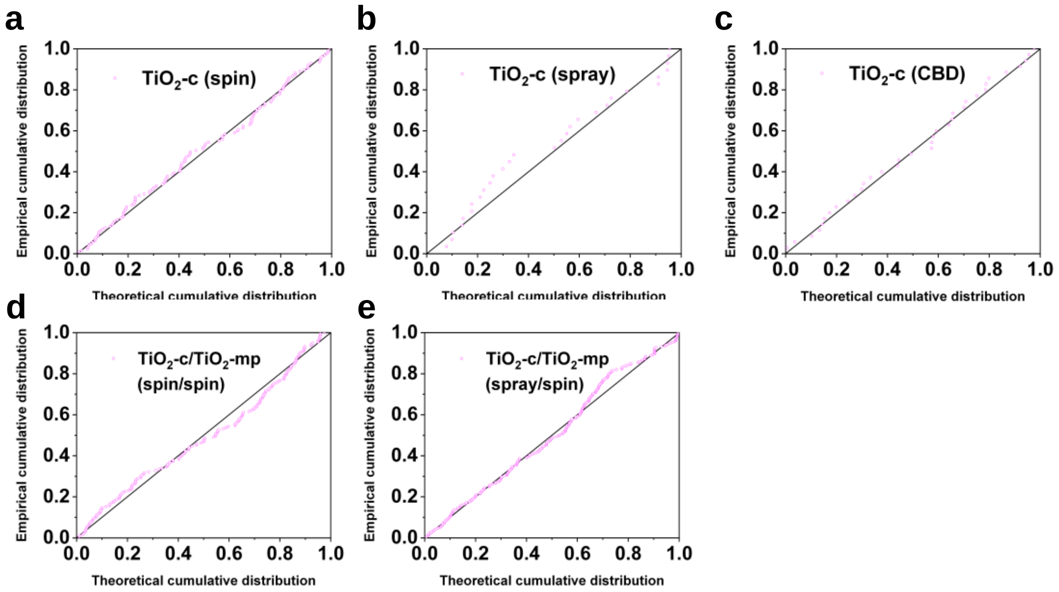

Supplementary Fig. 15 The normal probability plots of log(T_{S80m}) values for devices with different ETLs without encapsulation. **a** TiO_2-c by spin-coating. **b** TiO_2-c by spray-pyrolysis. **c** TiO_2-c by CBD. **d** TiO_2-c/ TiO_2-mp by spin-coating/ spin-coating. **e** TiO_2-c/ TiO_2-mp by spray-pyrolysis/ spin-coating.

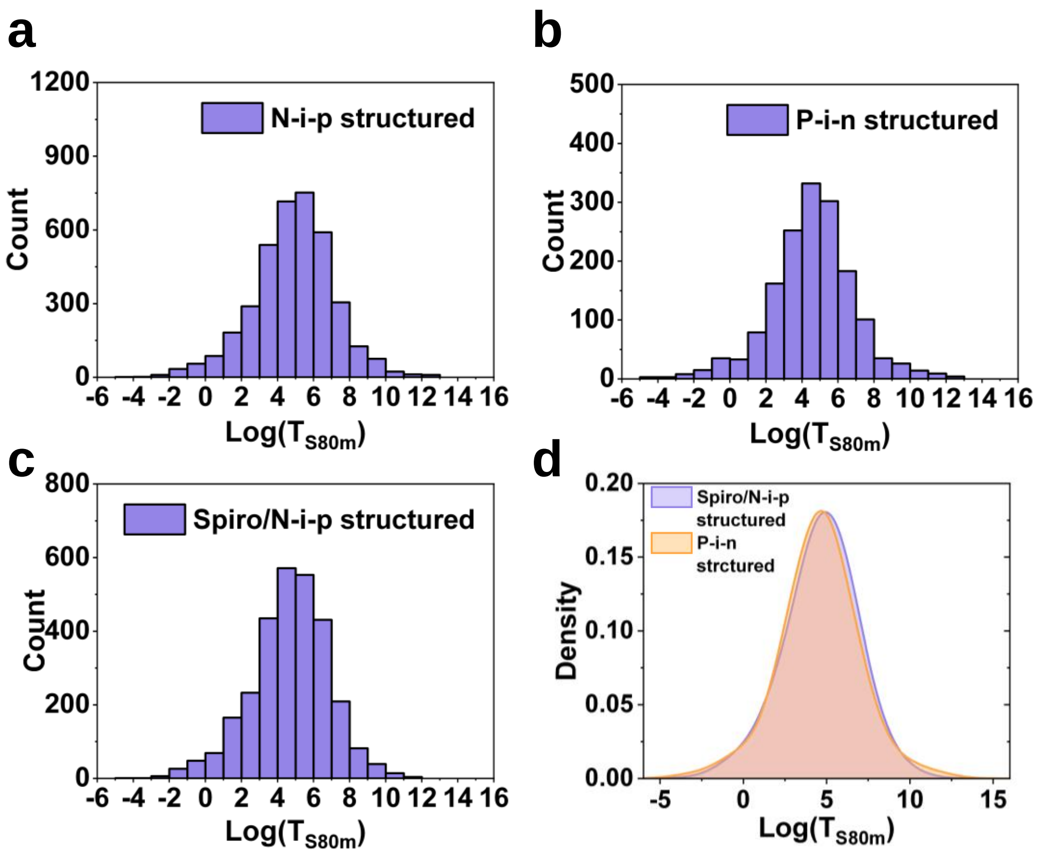

Supplementary Fig. 16 The histograms and kernel density estimation of log(T_{S80m}) values for devices with different device structures without encapsulation. **a** n-i-p structured devices. **b** p-i-n structured devices. **c** spiro-based n-i-p structured devices. **d** kernel density estimation.

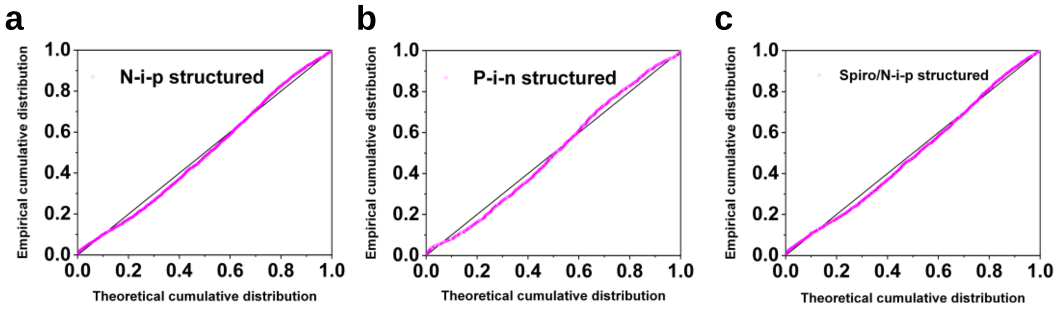

Supplementary Fig. 17 The normal probability plots of log(T_{S80m}) values for devices with different device structures without encapsulation. **a** n-i-p structured devices. **b** p-i-n structured devices. **c** spiro-based n-i-p structured devices.

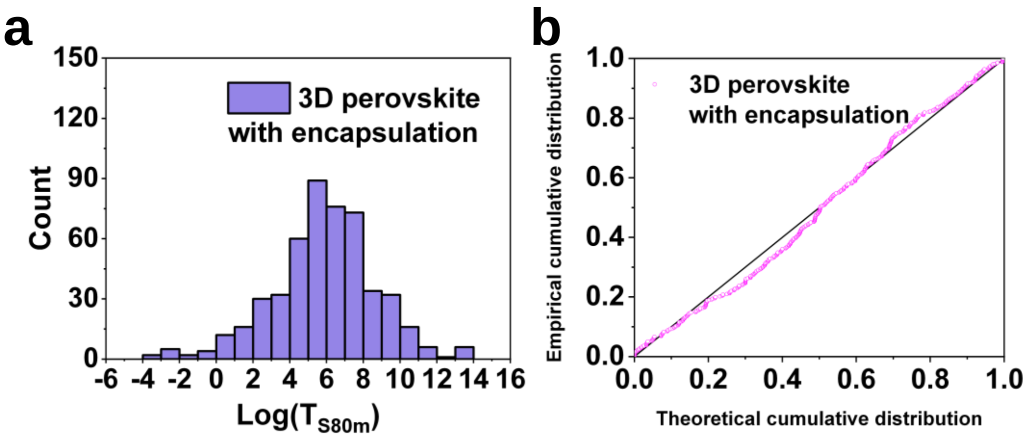

Supplementary Fig. 18 The distribution of log(T_{S80m}) values for 3D perovskite devices with encapsulation. **a** The histogram. **b** The corresponding normal probability plot.

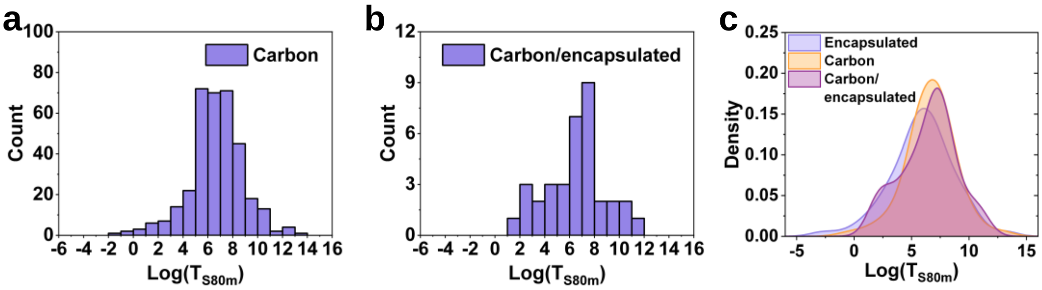

Supplementary Fig. 19 The histograms and kernel density estimation of log(T_{S80m}) values for carbon-based devices with and without encapsulation. **a** carbon-based devices without encapsulation. **b** with encapsulation. **c** kernel density estimation.

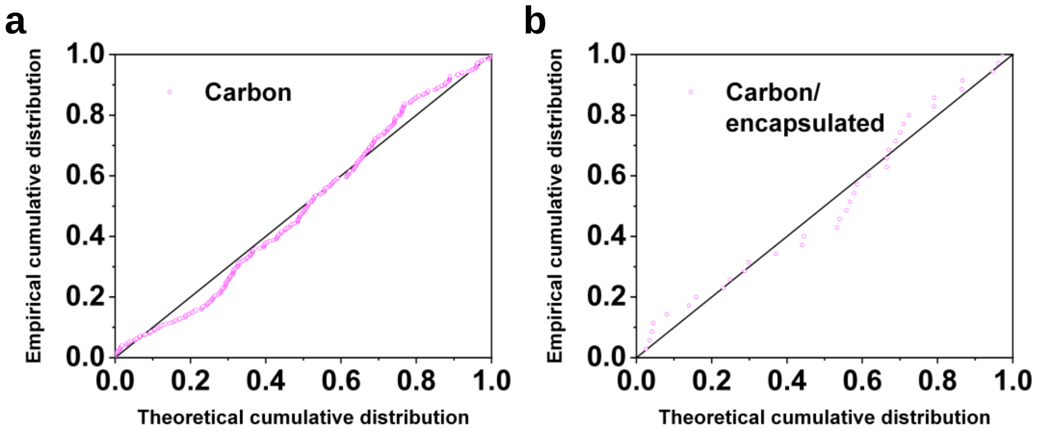

Supplementary Fig. 20 The normal probability plot of log(T_{S80m}) values for carbon-based devices with and without encapsulation. **a** carbon-based devices without encapsulation. **b** with encapsulation.

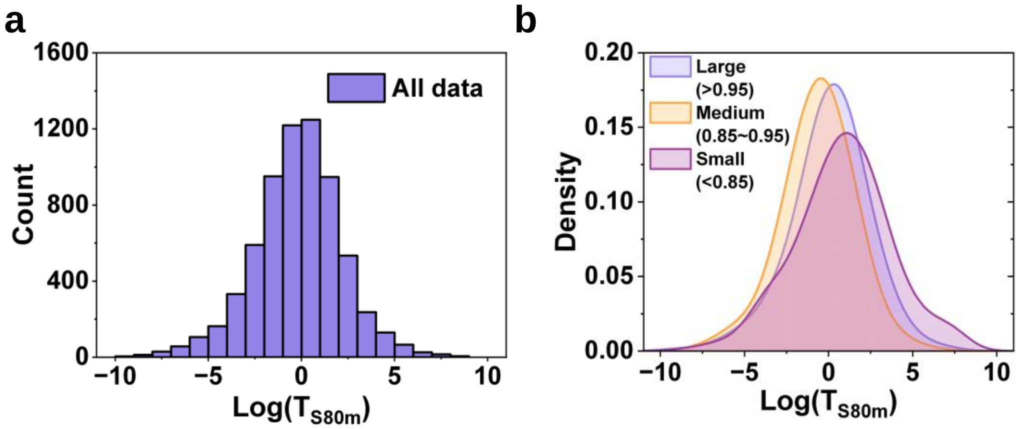

Supplementary Fig. 21 The distribution of all data with the reference conditions of 85 °C, 85% RH and 1 sun illumination. **a** Histogram of log(T_{S80m}) values for all data. **b** The kernel density estimation of the log(T_{S80m}) values for different tolerance factor regions of 3D perovskite devices without encapsulation.

Supplementary Tables

Supplementary Table 1. The availability of stability results.

**Available results**	**Counts**
T_{end} & E_{end}	7361
T_{95}	224
T_{S95}	23
T_{80}	1835
T_{S80}	88
T_{E80}	50
T_{SE80}	7
E_{1000h}	1009

Supplementary Table 2. Test conditions and structures of devices with top 10 T_{S80} values.

	T_{end} (h)	E_{end} (%)	T_{S80} (h)	T (°C)	RH (%)	I (mW cm^{-2})
1	11520	98.5	170086	30	50	0
	Structure: SLG/ITO/NiO-np/CsFAMAPbBrI/TiO_2-np/C_{60}; PCBM-60/Ag (encapsulated)					
2	6000	98	66271	25	65	0
	Structure: SLG/FTO/TiO_2-c/TiO_2-mp/ZrO_2-mp/MAPbI/Cu:NiO-np/Carbon					
3	5000	98	55226	25	0	0
	Structure: SLG/FTO/TiO_2-c/TiO_2-mp/Al_2O_3-mp/MAPbI/Carbon					
4	24000	90	50829	25	10	0
	Structure: SLG/FTO/TiO_2-c/TiO_2-mp/CsPbBr/Carbon					
5	4000	98	44180	25	40	0
	Structure: SLG/FTO/TiO_2-c/TiO_2-mp/MAPbI/Spiro-MeOTAD/Au					
6	5760	97.1	43675	27.5	50	0
	Structure: SLG/FTO/TiO_2-c/Al_2O_3-mp/NiO-np/FAMAPbI/Spiro-MeOTAD/Au					
7	7500	96	40996	25	50	0
	Structure: SLG/FTO/TiO_2-c/TiO_2-mp/FAMAPbBrI/PTAA/Au (encapsulated)					
8	3600	98	39762	25	15	0
	Structure: SLG/FTO/TiO_2-c/TiO_2-mp/MAPbI/Spiro-MeOTAD/Au					
9	7200	96	39357	30	50	0
	Structure: SLG/FTO/$SrTiO_3$/Graphene; Al_2O_3-mp/MAPbI/Spiro-MeOTAD/Au					
10	4800	97	35164	25	80	0
	Structure: SLG/FTO/TiO_2-c/TiO_2-mp/CsPbBr/Carbon					

Supplementary Table 3. Test conditions and structures of devices with top 10 T_{S80m} values.

	T_{end} (h)	E_{end} (%)	T_{S80} (h)	T (°C)	A_{tem}	RH (%)	A_{hum}	I (mW cm^{-2})	A_{light}	T_{S80m} (h)
1	1000	94.7	4097	85	43.28	85	4.25	100	1	753734
	Structure: SLG/FTO/TiO_2-c/FAMAPbBrI/PCPD2FBT:BCF/PEDOT:PSS/ITO/SLG (encapsulated)									
2	1000	94.2	3734	85	43.28	85	4.25	100	1	686954
	Structure: SLG/FTO/TiO_2-c/FAMAPbBrI/PCPD2FBT:BCF/PEDOT:PSS/ITO/SLG (encapsulated)									
3	1000	94	3606	100	94.77	40	2	100	1	683513
	Structure: SLG/FTO/TiO_2-c/CsPbBr/Carbon									
4	1500	90	3176	90	56.61	70	3.5	100	1	629413
	Structure: SLG/FTO/NiO-c/MAPbI/PCBM-60/PEI/Ag (encapsulated)									
5	1000	93.6	3374	85	43.28	85	4.25	100	1	620587
	Structure: SLG/FTO/TiO_2-c/FAMAPbBrI/PCPD2FBT:BCF/PEDOT:PSS/ITO/SLG (encapsulated)									
6	1000	92.5	2862	85	43.28	85	4.25	100	1	526484
	Structure: SLG/FTO/TiO_2-c/FAMAPbBrI/PCPD2FBT:BCF/PEDOT:PSS/ITO/SLG (encapsulated)									
7	1000	92	2676	85	43.28	85	4.25	100	1	492261
	Structure: SLG/FTO/TiO_2-c/CsFAMAPbBrI/PTAA/Au (encapsulated)									
8	500	95	2175	85	43.28	85	4.25	100	1	400106
	Structure: SLG/FTO/TiO_2-c/TiO_2-mp/CsPbBr/Carbon									
9	1000	90	2117	85	43.28	85	4.25	100	1	389572
	Structure: SLG/ITO/Spiro-TTB/CsFAPbBrI/LiF/C_{60}/SnO_2/ITO/Ag (encapsulated)									
10	1000	92.65	2922	100	94.77	25	1.25	100	1	346247
	Structure: SLG/FTO/TiO_2-c/TiO_2-mp/CsPbBrI/P3HT/Au									

Supplementary Table 4. Ion sizes used for tolerance factor calculation.

Positions	Ions	Sizes (pm)
A	Cs^+	167
	FA^+	253
	MA^+	217
B	Pb^{2+}	119
	Sn^{2+}	112
X	Br^-	196
	Cl^-	181
	I^-	220

Supplementary Table 5. Tolerance factors and counts of the most widely used 10 3D perovskite compositions.

	Compositions	Tolrance factors	Counts
1	$MAPbI_3$	0.9115	3618
2	$FA_{0.85}MA_{0.15}PbBr_{0.45}I_{2.55}$	0.9782	301
3	$Cs_{0.05}FA_{0.79}MA_{0.16}PbBr_{0.51}I_{2.49}$	0.9688	273
4	$CsPbBrI_2$	0.8096	198
5	$CsPbBr_3$	0.8149	124
6	$Cs_{0.05}FA_{0.81}MA_{0.14}PbBr_{0.45}I_{2.55}$	0.9699	103
7	$FAPbI_3$	0.9866	87
8	$CsPbI_3$	0.8072	70
9	$FA_{0.83}MA_{0.17}PbBr_{0.51}I_{2.49}$	0.9771	65
10	$CsPbBr_2I$	0.8122	44

Supplementary Table 6. Statistical results of 3D perovskite devices with large, medium and small tolerance factors.

Group	Sample	Average	Variance	Size	H_0 accepted	μ	T_A/T_B ratio
1	Large (>0.95)	5.300	5.362	1691	Yes	0.619	1.858
	Medium (0.85~0.95)	4.574	4.609	3608			
2	Small (<0.85)	6.049	8.097	402	Yes	1.282	3.605
	Medium (0.85~0.95)	4.574	4.609	3608			

Supplementary Table 7. Statistical results of 3D perovskite, 3D perovskite with 2D capping layer and 2D perovskite devices.

Group	Sample	Average	Variance	Size	H_0 accepted	μ	T_A/T_B ratio
1	2D perovskite	5.795	4.394	264	Yes	0.661	1.936
	3D perovskite	4.898	5.266	5755			
2	3D perovskite with 2D capping layer	5.941	6.080	65	Yes	0.572	1.771
	3D perovskite	4.898	5.266	5755			

Supplementary Table 8. Statistical results of Spiro-MeOTAD, PTAA, and P3HT devices.

Group	Sample	Average	Variance	Size	H_0 accepted	μ	T_A/T_B ratio
1	PTAA	4.967	7.388	161	No	-	-
	Spiro-MeOTAD	4.731	4.690	2930			
2	P3HT	5.267	10.30	128	Yes	0.206	1.228
	Spiro-MeOTAD	4.731	4.690	2930			

Supplementary Table 9. Statistical results of doped organic HTL, undoped organic HTL, inorganic HTL, HTL/carbon and HTL-free/carbon devices.

Group	Sample	Average	Variance	Size	H_0 accepted	μ	T_A/T_B ratio
1	Undoped organic HTL	5.028	4.668	341	Yes	0.043	1.044
	Doped organic HTL	4.775	5.036	3074			
2	Inorganic HTL	5.854	7.219	57	Yes	0.583	1.791
	Doped organic HTL	4.775	5.036	3074			
3	HTL/Carbon	7.132	5.745	94	Yes	1.969	7.163
	Doped organic HTL	4.775	5.036	3074			
4	HTL-free/Carbon	6.458	4.261	257	Yes	1.444	4.240
	Doped organic HTL	4.775	5.036	3074			

Supplementary Table 10. The balance between efficiency and stability of doped organic HTL, undoped organic HTL, inorganic HTL, HTL/carbon and HTL-free/carbon devices.

Sample	Efficiency (%)	Efficiency gain	Stability gain (T_A/T_B ratio)	Output gain
Doped organic HTL	17.90	1	1	1
Undoped organic HTL	17.10	0.955	1.044	0.997
Inorganic HTL	13.47	0.753	1.791	1.349
HTL/Carbon	9.944	0.556	7.163	3.983
HTL-free/Carbon	10.50	0.587	4.240	2.489

Supplementary Table 11. Statistical results of TiO_2-c, TiO_2-c/TiO_2-mp, SnO_2-c, SnO_2-np and other ETL devices.

Group	Sample	Average	Variance	Size	H_0 accepted	μ	T_A/T_B ratio
1	TiO_2-c /TiO_2-mp	4.957	8.187	413	Yes	0.447	1.563
	TiO_2-c	4.089	11.17	212			
2	SnO_2-c	4.722	7.131	78	No	-	-
	TiO_2-c	4.089	11.17	212			
3	SnO_2-np	5.619	7.021	105	Yes	0.914	2.494
	TiO_2-c	4.089	11.17	212			
4	Other ETLs	4.649	6.650	87	No	-	-
	TiO_2-c	4.089	11.17	212			

Supplementary Table 12. Statistical results of TiO_2-c (spin), TiO_2-c (spray), TiO_2-c (CBD), TiO_2-c/ TiO_2-mp (spin/spin), TiO_2-c/ TiO_2-mp (spray/spin).

Group	Sample	Average	Variance	Size	H_0 accepted	μ	T_A/T_B ratio
1	TiO_2-c (spray)	4.200	24.39	29	No	-	-
	TiO_2-c (spin)	3.903	9.424	127			
2	TiO_2-c (CBD)	5.425	10.34	35	Yes	0.542	1.719
	TiO_2-c (spin)	3.903	9.424	127			
3	TiO_2-c/ TiO_2-mp (spin/spin)	4.447	7.829	177	No	-	-
	TiO_2-c (spin)	3.903	9.424	127			
4	TiO_2-c/ TiO_2-mp (spray/spin)	5.390	7.998	233	Yes	0.956	2.601
	TiO_2-c (spin)	3.903	9.424	127			

Supplementary Table 13. Statistical results of n-i-p and p-i-n structured devices.

Group	Sample	Average	Variance	Size	H_0 accepted	μ	T_A/T_B ratio
1	N-i-p structured w/o encapsulation	4.879	4.921	3808	Yes	0.211	1.235
	P-i-n structured w/o encapsulation	4.557	5.390	1596			
2	Spiro-based N-i-p structured w/o encapsulation	4.692	4.576	2887	Yes	0.021	1.022
	P-i-n structured w/o encapsulation	4.557	5.390	1596			

Supplementary Table 14. Test conditions and structures of n-i-p structured devices without encapsulation with top 10 T_{S80m} values.

	T_{end} (h)	E_{end} (%)	T_{S80} (h)	T (°C)	A_{tem}	RH (%)	A_{hum}	I (mW cm^{-2})	A_{light}	T_{S80m} (h)
1	1000	92.65	2923	100	94.77	25	1.25	100	1	346247
	Structure: SLG/FTO/TiO_2-c/TiO_2-mp/CsPbBrI/P3HT/Au									
2	1000	92.44	2839	100	94.77	25	1.25	100	1	336252
	Structure: SLG/FTO/TiO_2-c/TiO_2-mp/CsPbBrI/P3HT/Au									
3	1000	91.20	2422	100	94.77	25	1.25	100	1	286955
	Structure: SLG/FTO/TiO_2-c/TiO_2-mp/CsPbBrI/P3HT/Au									
4	1000	86.44	1531	85	43.28	85	4.25	100	1	281674
	Structure: SLG/FTO/TiO_2-c/FAMAPbBrI/PCPD2FBT:BCF/PEDOT:PSS/ITO/SLG									
5	1000	90.02	2122	100	94.77	25	1.25	100	1	251411
	Structure: SLG/FTO/TiO_2-c/TiO_2-mp/CsPbBrI/P3HT/Au									
6	1000	84	1280	85	43.28	85	4.25	100	1	235415
	Structure: SLG/FTO/TiO_2-c/FAMAPbBrI/PCPD2FBT:BCF/PEDOT:PSS/ITO/SLG									
7	1000	81	1059	85	43.28	85	4.25	100	1	194786
	Structure: SLG/FTO/TiO_2-c/FAMAPbBrI/PCPD2FBT:BCF/PEDOT:PSS/ITO/SLG									
8	1000	89.9	2096	85	43.28	40	2	100	1	181414
	Structure: SLG/FTO/TiO_2-c/TiO_2-mp/CsPbBrI/P3HT/Au									
9	1000	79	982	85	43.28	85	4.25	100	1	180631
	Structure: SLG/FTO/TiO_2-c/FAMAPbBrI/PCPD2FBT:BCF/PEDOT:PSS/ITO/SLG									
10	1000	85.54	1429	100	94.77	25	1.25	100	1	169240
	Structure: SLG/FTO/ TiO_2-c/TiO_2-mp/CsPbBrI/P3HT/Au									

Supplementary Table 15. Test conditions and structures of p-i-n structured devices without encapsulation with top 10 T_{S80m} values.

	T_{end} (h)	E_{end} (%)	T_{S80} (h)	T (°C)	A_{tem}	RH (%)	A_{hum}	I (mW cm^{-2})	A_{light}	T_{S80m} (h)
1	960	90	2033	120	245.5	60	3	0	0.2	298775
	Structure: SLG/ITO/NiO/PS/MAPbI/PS/PCBM-60/Ag									
2	1100	95	4785	75	24.72	50	2.5	100	1	295762
	Structure: SLG/FTO/NiO-c/BMIMBF4/CsFAMAPbBrI/PCBM-60/BCP/Cr/Au									
3	1000	90	2118	100	94.77	20	1	100	1	200704
	Structure: SLG/ITO/PEDOT:PSS/FAMAPbBrI/NDI-ID(RR)/Al									
4	6000	75	4654	85	43.28	85	4.25	0	0.2	170806
	Structure: SLG/ITO/PEDOT:PSS/MAPbI/P(NDI2OD-T2)/Ag									
5	1000	95	4350	85	43.28	85	4.25	0	0.2	159663
	Structure: SLG/FTO/NiO-c/FAMAPbBrI/g-C3N4/PCBM-60; Graphene/BCP/Ag									
6	960	82	1079	120	245.5	60	3	0	0.2	158624
	Structure: SLG/ITO/NiO/PS/MAPbI/PCBM-60/Ag									
7	1000	83	1500	100	94.77	20	1	100	1	142148
	Structure: SLG/ITO/PEDOT:PSS/FAMAPbBrI/NDI-ID(RS)/Al									
8	960	80	960	120	245.5	60	3	0	0.2	141071
	Structure: SLG/ITO/NiO/PS/MAPbI/PCBM-60/Ag									
9	1000	95	4350	60	10.02	40	2	100	1	87185
	Structure: SLG/FTO/NiO-c/ FAMAPbBrI/g-C3N4/PCBM-60; Graphene/BCP/Ag									
10	500	86.7	782	85	43.28	50	2.5	100	1	84589
	Structure: SLG/ITO/NiO-c/CsMAPbI/PCBM-60/BCP/AZO-c/Ag									

Supplementary Table 16. Test conditions and structures of spiro-MeOTAD-based n-i-p structured devices without encapsulation with top 10 T_{S80m} values.

	T_{end} (h)	E_{end} (%)	T_{S80} (h)	T (°C)	A_{tem}	RH (%)	A_{hum}	I (mW cm^{-2})	A_{light}	T_{S80m} (h)
1	720	75.5	572	90	56.61	50	2.5	100	1	80903
	Structure: SLG/FTO/TiO_2-c/MAPbBr/Spiro-MeOTAD/Au									
2	720	90	1525	90	56.61	90	4.5	0	0.2	77504
	Structure: SLG/FTO/TiO_2-c/MAPbBr/Spiro-MeOTAD/Au									
3	700	70	438	120	245.5	30	1.5	30	0.43	69428
	Structure: PET/ITO/Nb_2O_5/CsPbBrI/Spiro-MeOTAD/Au									
4	720	59.5	309	90	56.61	50	2.5	100	1	43792
	Structure: SLG/FTO/TiO_2-c/MAPbBr/Spiro-MeOTAD/Au									
5	720	56.9	285	90	56.61	50	2.5	100	1	40322
	Structure: SLG/FTO/TiO_2-c/MAPbBr/Spiro-MeOTAD/Au									
6	500	90	1059	100	94.77	40	2	0	0.2	40046
	Structure: SLG/FTO/TiO_2-c/MAPbBr/Spiro-MeOTAD/Au									
7	2500	80	2500	50	5.238	60	3	100	1	39286
	Structure: SLG/ITO/SnO_2-c/PCBM-60/CsFAMAPbBrI/Spiro-MeOTAD/Au									
8	1096	80	1096	80	32.84	0	1	100	1	35993
	Structure: SLG/FTO/TiO_2-c/TiO_2-mp/CsFAPbBrI/Spiro-MeOTAD/Au									
9	1008	92	2698	25	0.855	50	2.5	1000	5	28916
	Structure: SLG/FTO/TiO_2-c/MAPbI/Spiro-MeOTAD/Ag									
10	2640	89.1	5104	65	13.66	40	2	0	0.2	27827
	Structure: SLG/FTO/TiO_2-c/TiO_2-mp/FAMAPbBrI/Spiro-MeOTAD/Au									

Supplementary Table 17. Statistical results of 3D perovskite devices without and with encapsulation.

Group	Sample	Average	Variance	Size	H_0 accepted	μ	T_A/T_B ratio
1	With encapsulation	5.896	7.659	496	Yes	0.935	2.546
	W/o encapsulation	4.784	5.080	5407			

Supplementary Table 18. Statistical results of encapsulated, carbon-based unencapsulated and carbon-based encapsulated devices.

Group	Sample	Average	Variance	Size	H_0 accepted	μ	T_A/T_B ratio
1	Carbon-based unencapsulated	6.639	4.732	351	Yes	0.451	1.569
	Encapsulated	5.896	7.659	496			
2	Carbon-based encapsulated	6.484	5.741	35	No	-	-
	Carbon-based unencapsulated	6.639	4.732	351			
3	Carbon-based encapsulated	6.484	5.741	35	No	-	-
	Encapsulated	5.896	7.659	496			

Supplementary Table 19. Statistical results of doped organic HTL, inorganic HTL and HTL/Carbon devices with different E_a values.

E_a (eV)	Sample	Average	Variance	Size	H_0 accepted	μ	T_A/T_B ratio
0	Inorganic HTL	5.071	5.754	57	Yes	0.078	1.081
	Doped organic HTL	4.515	4.717	3074			
0	HTL/Carbon	6.642	4.324	94	Yes	1.753	5.774
	Doped organic HTL	4.515	4.717	3074			
0.6	Inorganic HTL	5.854	7.219	57	Yes	0.583	1.791
	Doped organic HTL	4.775	5.036	3074			
0.6	HTL/Carbon	7.132	5.745	94	Yes	1.969	7.163
	Doped organic HTL	4.775	5.036	3074			
1.2	Inorganic HTL	6.636	14.02	57	Yes	0.967	2.630
	Doped organic HTL	5.036	8.193	3074			
1.2	HTL/Carbon	7.621	11.15	94	Yes	2.090	8.084
	Doped organic HTL	5.036	8.193	3074			

Supplementary Table 20. Statistical results of doped organic HTL, inorganic HTL, and HTL/Carbon devices with different γ values.

γ	Sample	Average	Variance	Size	H_0 accepted	μ	T_A/T_B ratio
0.5	Inorganic HTL	5.161	7.219	57	Yes	0.583	1.791
	Doped organic HTL	4.082	5.036	3074			
0.5	HTL/Carbon	6.439	5.745	94	Yes	1.969	7.163
	Doped organic HTL	4.082	5.036	3074			
1	Inorganic HTL	5.854	7.219	57	Yes	0.583	1.791
	Doped organic HTL	4.775	5.036	3074			
1	HTL/Carbon	7.132	5.745	94	Yes	1.969	7.163
	Doped organic HTL	4.775	5.036	3074			
1.5	Inorganic HTL	6.259	7.219	57	Yes	0.583	1.791
	Doped organic HTL	5.181	5.036	3074			
1.5	HTL/Carbon	7.537	5.745	94	Yes	1.969	7.163
	Doped organic HTL	5.181	5.036	3074			

Supplementary Table 21. Statistical results of 3D perovskite devices with large, medium, and small tolerance factors with 85 °C, 85% RH and 1 sun illumination as the reference conditions.

Group	Sample	Average	Variance	Size	H_0 accepted	μ	T_A/T_B ratio
1	Large (>0.95)	0.087	5.362	1691	Yes	0.619	1.858
	Medium (0.85~0.95)	-0.639	4.609	3608			
2	Small (<0.85)	0.838	8.097	402	Yes	1.282	3.605
	Medium (0.85~0.95)	-0.639	4.609	3608			

Supplementary References

1 Roesch, R. *et al.* Procedures and Practices for Evaluating Thin-Film Solar Cell Stability. *Advanced Energy Materials* **5** (2015).

2 Haillant, O., Dumbleton, D. & Zielnik, A. An Arrhenius approach to estimating organic photovoltaic module weathering acceleration factors. *Solar Energy Materials and Solar Cells* **95**, 1889-1895 (2011).
